# Study on the Gamma Irradiation Characteristics of a Carbon Nanotube Sponge/Polydimethylsiloxane/Tungsten Oxide Flexible Force-Sensitive Structure

**DOI:** 10.3390/mi13071024

**Published:** 2022-06-28

**Authors:** Xingcheng Han, Xin Li, Ruirong Wang, Jinping Liu, Lishuang Liu

**Affiliations:** 1 Key Laboratory of Instrumentation Science and Dynamic Measurement, Shanxi Province Key Laboratory of Quantum Sensing and Precision Measurement, North University of China, Taiyuan 030051, China; zbhanxc@nuc.edu.cn (X.H.); lixinxjx@163.com (X.L.); wangruirong@yeah.net (R.W.); s1906033@163.com (J.L.); 2 Department of Electronic Engineering, Taiyuan Institute of Technology, Taiyuan 030008, China

**Keywords:** gamma irradiation, force-sensitive structure, CNT sponge/PDMS/WO_3_

## Abstract

This paper proposes a new type of flexible force-sensitive structure that is resistant to gamma radiation and is made of tungsten oxide (WO_3_) powder, polydimethylsiloxane (PDMS), and carbon nanotube (CNT) sponge. The thickness of the sample was 2.2 mm, the middle interlayer was composed of a carbon nanotube (CNT) sponge and PDMS to form a conductive layer, and the upper and lower layers were made of tungsten oxide and PDMS, which formed a gamma-ray shielding layer. When the particle size of the tungsten oxide powder was 50 nm, 100 nm, and 1 µm, the composite force-sensitive structure exhibited better force-sensitive performance. The composite force-sensitive structure was irradiated with doses of 5, 20, 50, and 100 KGy through 60Co- rays with an energy of 1.25 MeV. The results showed that the force-sensitive characteristics changed little in significance after irradiation by different doses of gamma rays, indicating that the force-sensitive structure has good resistance to gamma radiation. This flexible stress sensor can be used in soft robots and health inspection, even in harsh environments without significant performance loss.

## 1. Introduction

Flexible stress sensors are devices that can convert stress or strain signals into electrical signal changes. They have a wide range of applications [1,2,3,4,5], including health detection, motion detection, and soft robotics, but when they are used in medical, aerospace, and other irradiated environments, they can cause irradiated thermal damage and degrade their performance [6,7,8,9,10]. Therefore, further research is needed to improve the adaptability of flexible stress sensors in different environments. Ke Liu et al. modified PDMS/CNT composites by irradiating them with a CO_2_ laser to form a superhydrophobic surface [11]. This strain sensor can be used to detect human movement and physiological signals in harsh environments. Lianhui Li et al. prepared a type of multifunctional superhydrophobic flexible stress sensor with Multi-walled carbon nanotubes and thermoplastic elastomer (MWCNT/TPE) composite material, which had strong waterproof, acid, and alkali resistance, and could work in wet environments and corrosion conditions [12]. However, most of the research on flexible sensors is based on superhydrophobicity, biodegradability, and reproducibility [13,14]. Therefore, it is necessary to prepare a force-sensitive structure with antiradiation properties. 

Heavy metals [15,16,17,18], such as lead, tungsten, and iron, are commonly used as gamma-ray shielding materials, but such metal powders affect the conductive network of force-sensitive sensors. A large number of studies have proved that composites containing these heavy metal elements have certain gamma shielding properties. The results [19,20] showed that tungsten oxide has good gamma-ray shielding ability, and the linear attenuation coefficient increases with the increase in tungsten oxide content. Therefore, in this experiment, tungsten oxide powder was used to achieve gamma-ray shielding. Tungsten oxide does not directly affect the conductive network, and its particles can fix the conductive network to ensure the stability of the electrical properties of the composite. In order to obtain better shielding performance, this paper also designs the structure to perform ray shielding in all directions. Compared with direct mixed doping, the sandwich structure can shield gamma rays better.

This paper proposes a force-sensitive structure that is resistant to gamma irradiation and is made of tungsten oxide powder, PDMS, and CNT sponge to create a sandwich structure. Tungsten oxide powder was used to resist gamma rays, the CNT sponge provided a conductive network, and PDMS was used as the flexible substrate. The composite structure showed good force-sensitivity, and the mixtures prepared using three different sizes of tungsten oxide particles all showed better force-sensitivity than the CNT sponge/PDMS. The attenuation of the CNT sponge/PDMS/WO_3_ was lower than that of the CNT sponge/PDMS sample after different doses of γ irradiation, indicating that the structure had good radiation resistance. Due to the fact of its light weight, good flexibility, easy preparation, and good shielding ability, the composite material provides further development ideas for flexible wearable devices and increases the application scenarios of flexible wearable devices.

## 2. Experimental

### 2.1. Materials

The carbon nanotube (CNT) sponge was purchased from Jiangsu Xianfeng Nano Material Technology Co., Ltd, Nangjing, China. The inner diameter of the carbon tubes was 10–20 nm, the outer diameter was 30–50 nm, the porosity was 99%, and the density was 10 mg/cm^3^. The carbon nanotube sponge was a black block with a thickness of approximately 1.2 mm. Tungsten oxide (WO_3_) powder was purchased from Qinghe County Yuanyao Alloy Products Co., Ltd, Xingtai, China. The color was light yellow. The particle sizes of the tungsten oxide used in the experiment were 50 nm, 100 nm, and 1 μm. Polydimethylsiloxane (PDMS) and the curing agent were purchased from Dow Corning, Midland, MI, USA.

### 2.2. Structure Preparation

**Figure 1:**
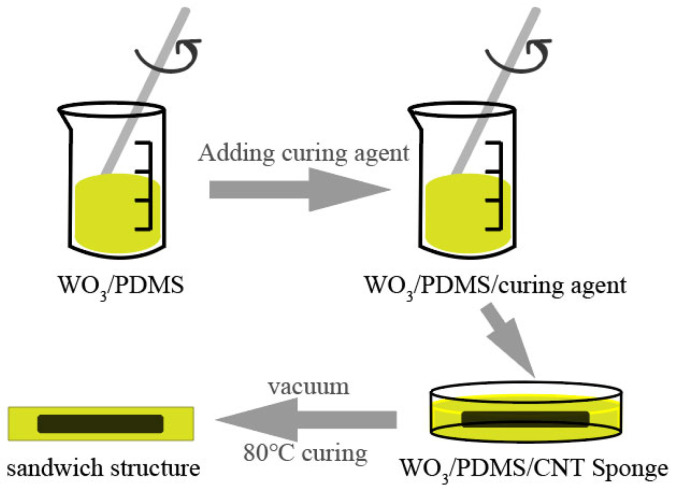
Flow chart of the force-sensitive structure’s processing and preparation.

**Figure 2:**
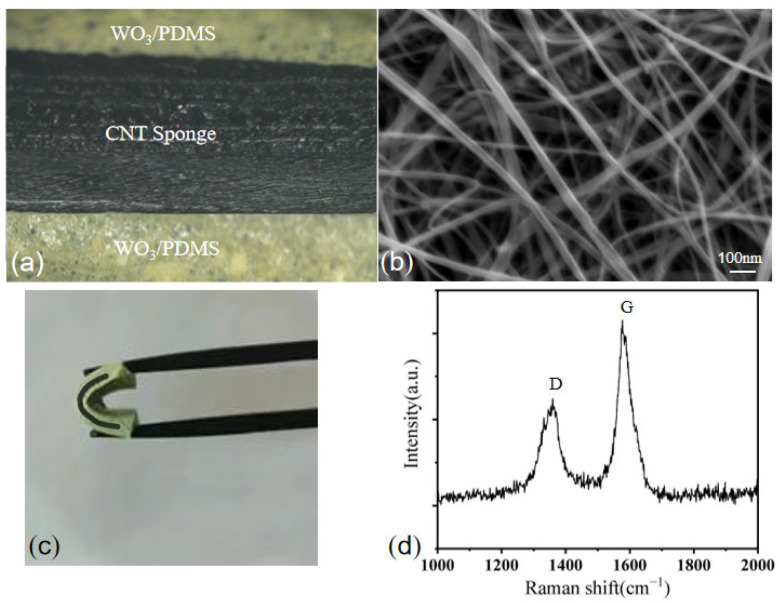
The CNT sponge/PDMS/WO_3_ force-sensitive structure: (**a**) cross-sectional view; (**b**) SEM image; (**c**) flexible display of physical objects; (**d**) Raman diagram of the CNT sponge.

The preparation process of the CNT sponge/PDMS/WO_3_ force-sensitive structure is shown in Figure 1. First, WO_3_:PDMS was mixed at a mass ratio of 1:1 and stirred evenly. Then, a certain amount of curing agent was added to the mixed solution and stirred again. Then, the CNT sponge was immersed in the mixed solution, and the position of the sponge was adjusted to keep it in the middle layer of the solution. It was allowed to stand for 12 h under vacuum to eliminate bubbles in the solution. After curing at 80 °C for 2 h, the preparation of the structure was complete. Figure 2a is a cross-sectional view of the force-sensitive structure. Studies have shown that the greater the content of the shielding material, the better the shielding effect [21,22]; however, it was found in the experimental operation that the higher the content of tungsten oxide, the worse the flexibility of the force-sensitive composite. In order to consider both the flexibility and radiation resistance, 50% WO_3_ doping was used. The tungsten oxide particles used in the experiment had three different particle sizes of 50 nm, 100 nm, and 1 μm. In the experiment, three force-sensitive structures with different particle sizes and a 50% mass fraction of WO_3_ were prepared. The CNT sponge/PDMS was also prepared for comparison with samples containing WO_3_.

**Figure 3:**
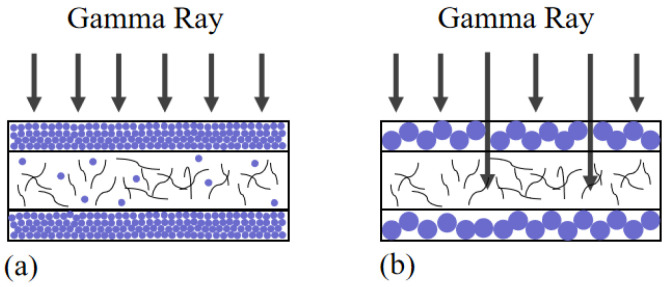
Schematic diagram of the sandwich structure: (**a**) nanometer-sized WO_3_ particles (small particles); (**b**) micron-sized WO_3_ particles (large particles).

Many radiation shielding studies have shown that the greater the thickness of a sample, the better the shielding effect at the same powder content [22,23]. However, increasing the thickness of a material will decrease its flexibility, so an appropriate thickness should be selected. Figure 3 shows a schematic diagram of the prepared sandwich structure. The thickness of the middle conductive interlayer was approximately 1.2 mm, the thickness of the upper and lower gamma shielding layers was approximately 0.5 mm each, and the overall thickness was approximately 2.2 mm. The carbon nanotube sponge was soaked in the solution to allow it to gradually penetrate the sponge and fill the gaps. When the particle size of the WO_3_ powder was small enough to penetrate into the sponge, WO_3_ entered the middle conductive layer, as shown in Figure 3a. It was observed in the experiment that when the particle size of WO_3_ was 50 nm, a small amount of WO_3_ powder entered the CNT sponge. The powder with a larger particle did not enter the middle layer and formed the structure shown in Figure 3b. The real figure prepared in the experiment is shown in Figure 2a, which is a cross-sectional view of the sample. The tungsten oxide powder used in the experiment contained impurities, causing some black dots to appear on the shielding layer. An SEM image of the CNT sponge is shown in Figure 2b. The physical object is shown in Figure 2c, which demonstrates the good flexibility of the sample by bending it. Raman spectroscopy was used to characterize the CNT sponge, and two characteristic peaks were found, namely defect peak (D peak) of CNT and graphite peak (G peak).

### 2.3. Experimental Conditions

In order to study the anti-gamma ray properties of the force-sensitive structure, irradiation experiments were conducted on four samples. The experiment was carried out at the Chinese Academy of Radiation Protection, the radiation source was 60Co-γ rays, and the ray energy value was 1.25 MeV. The samples were placed in a glass bottle, and the maximum dose rate used in the experiment was 25 Gy/min. The samples were exposed to doses of 5, 20, 50, and 100 KGy. The test methods of the irradiation effect included an in situ test and a shift test. In situ testing means that the electrical properties of the samples are monitored in real time while irradiation is carried out. The shift test method was adopted here, that is, after the sample was irradiated to a certain dose, it was moved out of the irradiation chamber and then tested for electrical properties.

To prevent external conditions, such as the experimental environment, from affecting the force-sensitive performance, the force-sensitive test experiments were carried out on the same experimental platform and in the same environment. The multimeter was connected to a computer through a data cable, and the resistance change of each sample within the 50% stretching range was recorded in real time. The stretching length was controlled by an electric stage, and the stretching speed was set to 1 mm/s.

## 3. Test Results

### 3.1. Force-Sensitive Performance under Different WO_3_ Particle Sizes

**Figure 4:**
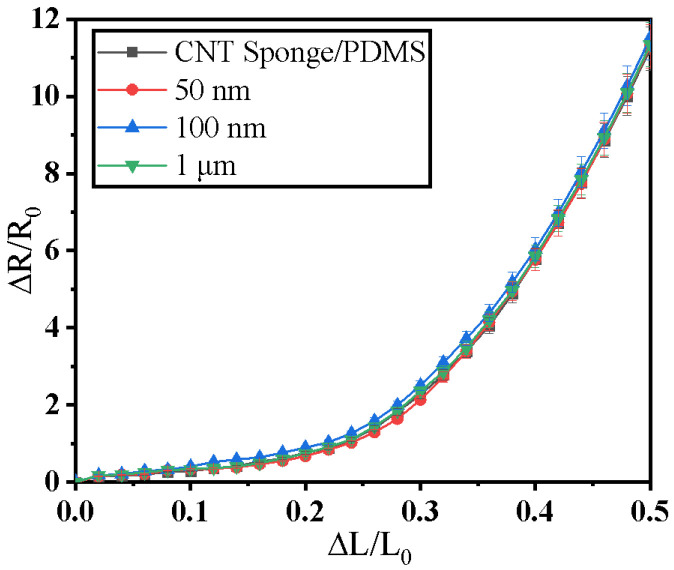
CNT sponge/PDMS/WO_3_ structure force-sensitive characteristic curves with different WO_3_ particle sizes.

In the experiment, the force-sensitive performance of the CNT sponge/PDMS/WO_3_ structure prepared by mixing WO_3_ with different particle sizes without irradiation was tested, and the results are shown in Figure 4. The force-sensitive properties of the sample without WO_3_, namely, the CNT sponge/PDMS in the figure, were also tested. It can be observed from the figure that when the particle size was different, the force-sensitive curves of the samples were nearly identical, indicating that the particle size of WO_3_ did not affect the force-sensitive characteristics of the structure.

### 3.2. Force-Sensitive Performance after Irradiation with Different Doses

**Figure 5:**
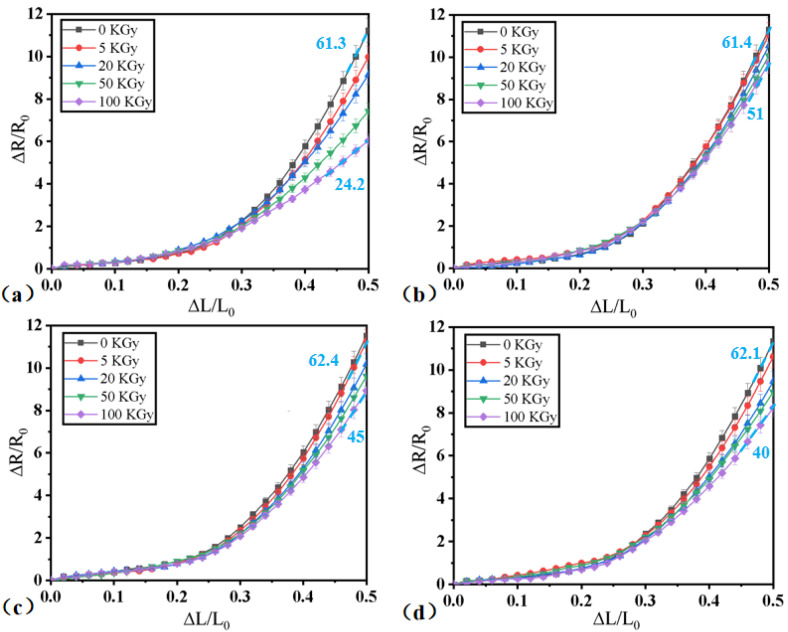
The force-sensitive characteristics of the structure after different doses of gamma irradiation; (**a**) the CNT sponge/PDMS and when the particle size of tungsten oxide was (**b**) 50 nm; (**c**) 100 nm; (**d**) 1 μm.

Irradiation experiments were carried out on four samples, and the irradiation dose was increased to 100 KGy. The force sensitivity of samples irradiated with different doses was tested again, and the test results are shown in Figure 5. The force-sensitive characteristic curves all showed the same trend. With the increase in the gamma radiation dose, the force sensitivity of the samples became worse and the sensitivity decreased.

Here, the sensitivity was defined as (ΔR/R_0_)/ε, where ε was ΔL/L_0_. As shown in Figure 5a, after gamma irradiation, the sensitivity of the CNT sponge/PDMS sample decreased from 61.3 to 24.2, a decrease of approximately 60%. The samples with tungsten oxide particles had low sensitivity attenuation after gamma irradiation. The sensitivity of the tungsten oxide samples with particle sizes of 50 nm, 100 nm, and 1 μm decreased by 17%, 28%, and 35.6%, respectively. As the WO_3_ particle size increased, fluctuations in the force-sensitive performance curves after irradiation with different doses also gradually increased. The larger the particle size of WO_3_, the greater the influence of gamma rays on the force-sensitive performance of the sample. J Kim et al. studied the influence of W particle size on the shielding effect, and the results showed that, when the photon energy was lower than 1 MeV, the smaller the particle size, the better the shielding effect. Here, the influence of the particle size on shielding performance was observed even at 1.25 MeV, possibly due to the differences in the experimental conditions [24]. As shown in Figure 3, when a smaller particle size of WO_3_ was used, more gaps were filled in the composite, which provided greater shielding against gamma rays. A smaller WO_3_ particle size increased the specific surface area, and more gamma loss interfaces were formed, which also reduced the influence of the gamma rays on the composite.

In this study, a new type of flexible stress sensor with gamma-ray shielding performance was proposed and compared with previous reports on flexible force-sensitive materials as shown in Table 1. The characteristic of the flexible force-sensitive structure prepared in this paper was that it had a certain gamma shielding performance.

## 4. Conclusions

This paper proposed a force-sensitive structure with good resistance to gamma irradiation, which was a sandwich structure made of WO_3_ powder, PDMS, and CNT sponge. Tungsten oxide powder was used for gamma-ray shielding, the CNT sponge provided a conductive network, and PDMS was used as a flexible substrate. The composite structure exhibited good force-sensitive properties. The force-sensitive structures prepared from three different WO_3_ particle sizes showed consistent force-sensitive properties, indicating that the tungsten oxide particle size did not affect the force-sensitive properties of the composite. Compared with the CNT sponge/PDMS sample, the sample with tungsten oxide particles had less attenuation of force sensitivity under irradiation, indicating that the designed structure had good radiation resistance. This experiment provides further development ideas for the application of flexible stress sensors in irradiation environments.

## Figures and Tables

**Table 1:** Multifunctional flexible stress sensing material.

Material	Function	Reference
Graphene-coated carbon nanotube aerogels	Resistance to creep and fatigue	[1]
Graphene foam (GF)/PDMS	Highly stretched and sensitive	[3]
Laser-irradiated PDMS/CNT composite	Superhydrophobic	[11]
MWCNT/TPE composite film coatings	Superhydrophobic smart coating	[12]
Hydrogels	Rapid self-healing	[13]
CNT sponge/PDMS/WO_3_	Gamma irradiation resistance	This work

## Data Availability

Not applicable.
